# Hospital patient safety situational analyses: Cameroon, Mali and Senegal

**DOI:** 10.1186/1753-6561-5-S6-P323

**Published:** 2011-06-29

**Authors:** SB Syed, V Djientcheu, NMD Badiane, L Bengaly, K Quevison, J Hightower, D Pittet

**Affiliations:** 1 WHO, Geneva, Switzerland; 2 Hôpital Central Yaoundé, Yaoundé , Cameroon; 3 University Hospital Fann, Dakar, Senegal; 4 CHU Hospital Gabriel Touré , Bamako, Mali; 5 WHO, Addis Ababa, Ethiopia; 6 University of Geneva Hospitals, Geneva, Switzerland

## Introduction / objectives

Hôpitaux Universitaires de Genève partners with hospitals in Cameroon, Mali & Senegal via African Partnerships for Patient Safety (APPS). Baseline hospital patient safety understanding is largely unknown in African settings. APPS is framed around 12 WHO AFRO action areas; prevention of health care-associated infection is a common platform.

## Methods

First, APPS developed a situational analysis tool at a cross-country workshop to define questions for systematic data collection on 12 patient safety action areas. Second, each APPS hospital formed teams to conduct analyses. Third, an external expert worked on-site with each team to validate findings. Finally, results were shared at a second cross-country Workshop, forming the basis for action.

## Results

Each hospital constructed a detailed patient safety profile. Key findings on infection prevention and control (IPC) were highlighted. First, although each hospital had reliable running water, two hospitals could not confirm a clean supply; no hospitals had access to alcohol based hand rub or single use towels and two hospitals did not have reliable soap supply. Second, IPC activities were in place in hospitals but with no full time IPC doctor or nurse. Third, hospital policies/guidelines existed for technical areas in each hospital. Fourth, capacity & systems for IPC surveillance was variable, routine notification of infectious disease in place in two hospitals. Fifth, no hospitals had antibiotic use policies. IPC findings defined APPS action.

## Conclusion

The tested tool can be used in African hospitals as a basis for patient safety action. This may be applicable to other developing world settings.

## Disclosure of interest

None declared.

